# Laser Capture Microdissection Protocol for Xylem Tissues of Woody Plants

**DOI:** 10.3389/fpls.2016.01965

**Published:** 2017-01-04

**Authors:** Olga Blokhina, Concetta Valerio, Katarzyna Sokołowska, Lei Zhao, Anna Kärkönen, Totte Niittylä, Kurt Fagerstedt

**Affiliations:** ^1^Viikki Plant Science Centre, Department of Biosciences, University of HelsinkiHelsinki, Finland; ^2^Plant Stress Signaling, Instituto Gulbenkian de CiênciaOeiras, Portugal; ^3^Umeå Plant Science Centre, Department of Forest Genetics and Plant Physiology, Swedish University of Agricultural SciencesUmeå, Sweden; ^4^Department of Plant Developmental Biology, Institute of Experimental Biology, University of WrocławWrocław, Poland; ^5^Viikki Plant Science Centre, Department of Agricultural Sciences, University of HelsinkiHelsinki, Finland

**Keywords:** cryosection, laser capture microdissection, ray cells, RNA integrity, tracheids, xylem fibers

## Abstract

Laser capture microdissection (LCM) enables precise dissection and collection of individual cell types from complex tissues. When applied to plant cells, and especially to woody tissues, LCM requires extensive optimization to overcome such factors as rigid cell walls, large central vacuoles, intercellular spaces, and technical issues with thickness and flatness of the sections. Here we present an optimized protocol for the laser-assisted microdissection of developing xylem from mature trees: a gymnosperm (Norway spruce, *Picea abies*) and an angiosperm (aspen, *Populus tremula*) tree. Different cell types of spruce and aspen wood (i.e., ray cells, tracheary elements, and fibers) were successfully microdissected from tangential, cross and radial cryosections of the current year’s growth ring. Two approaches were applied to achieve satisfactory flatness and anatomical integrity of the spruce and aspen specimens. The commonly used membrane slides were ineffective as a mounting surface for the wood cryosections. Instead, in the present protocol we use glass slides, and introduce a glass slide sandwich assembly for the preparation of aspen sections. To ascertain that not only the anatomical integrity of the plant tissue, but also the molecular features were not compromised during the whole LCM procedure, good quality total RNA could be extracted from the microdissected cells. This showed the efficiency of the protocol and established that our methodology can be integrated in transcriptome analyses to elucidate cell-specific molecular events regulating wood formation in trees.

## Introduction

Laser capture microdissection (LCM) was originally developed to assist semi-automatic collection of individual cells and tissues from complex biological specimens for subsequent genomic and transcriptomic analyses (Horneffer et al., 2007). The method allows precise analysis of target cells, and is contact- and contamination-free (Gautam and Sarkar, 2015). LCM is a microscope-based technique and involves several crucial steps: sample preparation (sample embedding and sectioning, or cryosectioning), visualization of intact cells or tissues to be isolated, outlining and laser-cutting of the selected areas and, finally, collection of dissected cells either by gravity into a tube, or by catapulting the target cells against gravity into an adhesive cap of a collection tube (Horneffer et al., 2007).

Development of new techniques for high-throughput data acquisition, concurrent development of bioinformatic analyses and increasing availability of sequenced genomes have made LCM a method of choice for elucidating the cell type-specific molecular features. Downstream applications can cover virtually every aspect of cell biology. Above all, gene expression analyses are, nowadays, often performed through genome-wide expression profiling based on microarray or next generation sequencing (NGS) technologies (whole genome sequencing, targeted genome sequencing, RNA sequencing and ribosome profiling). The combination of LCM with NGS techniques is a powerful approach to study cell- and tissue-specific processes (Abbott et al., 2010; Jyske et al., 2015).

Microdissection of plant tissues and individual cells poses a challenge due to the presence of a cell wall, a large central vacuole in most fully differentiated cells and, in some tissues, large intercellular spaces (Nakazono et al., 2003). The LCM technique, which was originally developed for animal tissues, has required substantial optimization for plant studies. Asano et al. (2002) were the first to apply LCM on plant tissues in a study of phloem-specific gene expression in rice (*Oryza sativa*). Extensive work was later performed to establish protocols on plant tissues, mainly on herbaceous species and by using paraffin-embedded material (Kerk et al., 2003; Inada and Wildermuth, 2005; Rajhi et al., 2011; Shiono et al., 2014). For example, pre-fixed paraffin sections of maize (*Zea mays*) roots were analyzed for differential gene expression between epidermal cells and vascular tissues using a microarray (Nakazono et al., 2003). The same experimental approach has been applied in studies on aerenchyma formation in the cortex of maize roots (paraffin sections on membrane slides) (Rajhi et al., 2011), and on suberinization of roots during formation of a barrier to radial oxygen loss in rice (Shiono et al., 2014). The findings of the Nakazono group on LCM of paraffin-embedded tissues have been summarized in a methodological article by Takahashi et al. (2010).

Non-paraffin embedding procedures have been introduced recently to LCM of plant tissues. An alternative strategy of sample preparation which guaranteed RNA quality and morphological integrity, replaces paraffin with Steedman’s wax. This polyester wax has a lower melting point than paraffin (38-40°C *vs* 60-65°C), and can be washed out by ethanol, an overall milder sample treatment than the paraffin removal by xylene. The Steedman’s wax-embedding has been performed, for example, in studies on arbuscular mycorrhiza and root cortical cells of barrel medic (*Medicago truncatula*) (Gomez et al., 2009; Hogekamp et al., 2011). Good results in terms of RNA quality and RNA sequencing have been observed also after incubation of plant material in polyethylene glycol (PEG)-ethanol mixture and subsequent inclusion in PEG-modified Steedman’s wax and polyethylene naphtalate (PEN)-coated slides for LCM (Roux et al., 2014). This protocol was developed further and 100% PEG was used as an embedding medium with sections mounted on PEN slides (Jardinaud et al., 2016). Much thicker longitudal root sections (35 μm) of barrel medic have been prepared for LCM using embedding in tissue-freezing medium and collected mycorrhizal material was used for gene expression profiling (Gaude et al., 2012).

LCM has also been used to study tissue- and cell-specific processes of non-xylem tissues in woody plants. Abbott and co-workers successfully isolated resin ducts and cambial tissues from tangential cryosections of 2-year-old white spruce (*Picea glauca*) plantlets. This allowed the characterization of RNA, determination of enzyme activity and terpenoid metabolites (Abbott et al., 2010). Stereo microscope-assisted dissection of cryosections followed by microgenomics has been used in a functional study of neighboring meristematic cells in the vascular cambium of *Populus* species (Goué et al., 2008, 2012). Jyske and co-workers (Jyske et al., 2015) have applied the LCM technology to assess the seasonal variation in the phloem structure and its constituents in mature Norway spruce (*Picea abies*) trees. LCM has been used also to investigate gene expression in phloem of Norway spruce (Nagy et al., 2014). In all these studies, the soft phloem, cambial or cortex tissues appeared to be suitable for preparation of samples through either cryosectioning or paraffin-embedding for LCM.

Xylem tissue with its highly lignified cell walls has proven challenging for LCM applications. Larisch et al. (2012) used LCM to separate developing and mature wood from twigs of poplar (*Populus* × *canescens*), and subsequently analyzed the mature wood transcriptome using whole-genome microarrays (Larisch et al., 2012). The authors argued that ray cells, as the main living cells in mature wood, contributed most to the isolated RNA. However, the method did not allow ray-cell-specific isolation of RNA due to problems with RNA degradation and yield. Recently, LCM was also used to prepare ray cell-wall-enriched material for lignin composition analysis from the wood of Amur cork tree (*Phellodendron amurense*) (Zheng et al., 2016). This analysis provided a cell specific lignin profile, but no RNA was isolated. The studies of Larisch et al. (2012) and Zheng et al. (2016) illustrate the potential of LCM method, while at the same time highlighting the need for a robust xylem-cell-specific LCM protocol which does not compromise RNA quality.

Here we developed a protocol for LCM of developing xylem tissues from 40-year-old Norway spruce (*Picea abies*) and aspen (*Populus tremula*) trees, representatives of gymnosperms and angiosperms. The work with the gymnosperm tree was done at the Viikki Plant Science Centre in Finland, and with the angiosperm tree at the Umeå Plant Science Centre in Sweden. The main goal was to achieve high quality RNA from different xylem cell types (ray parenchymal cells, xylem fibers and xylem tracheary elements (i.e., tracheids in spruce) suitable for high-throughput analyses. Special emphasis was placed on the isolation of ray parenchymal cells, while avoiding collecting ray tracheids typical for spruce. Slightly different approaches were found to be optimal for the preparation and handling of spruce and aspen samples prior to LCM. During preparation of the sections we tested varying sample thicknesses and orientations. We used PEN-coated slides and plain glass slides, choosing the latter as the best mounting surface to fix the cryosections. We established different dehydration procedures for Norway spruce and aspen. Correct handling of the samples prior to cryosectioning was crucial for the anatomical and RNA integrity. Several methodological problems were overcome during the method development. These included: difficulty in achieving sufficient flatness of the sections on slides, static electricity, very low RNA yield, and optimization of laser power for cutting specimens in cell wall-rich areas in comparison with the cytosolic part of the cells. These methodological issues together with some useful tips are addressed in the ‘critical notes’ section and in the troubleshooting table.

### Protocol Overview

We developed protocols for the LCM of wood sections for extraction of cell type-specific total RNA from developing xylem of Norway spruce and aspen. Disks of wood stems of 40-year-old spruce and aspen trees were collected during the active secondary growth and stored at -80°C. For an easier handling of the samples during preparation of the cryosections, the stem disks were further sawn into smaller blocks. The blocks were then used to prepare 30/40 μm thick tangential (spruce) or cross and radial (aspen) sections using a cryomicrotome. The cryosections, mounted on microscope glass slides, were fixed with ethanol. Spruce and aspen wood required different procedures for dehydration and flattening of the sections. Spruce tangential sections were flattened with a pipette tip during the last dehydration step in 100% ethanol. Aspen cross and radial sections were fixed in 75% ethanol and then flattened using a microscope slide sandwich during dehydration in a freeze-dryer chamber. Intrinsic differences in wood anatomy between gymnosperms and angiosperms determined the sectioning plane and, subsequently, the flattening method for the cryosections. For example, the presence of large vessels in aspen prevented the use of tangential sections, because of the high probability of section breakage along the vessel. Individual cell types (ray parenchymal cells, tracheids, and fibers) were isolated by LCM using a PALM Micro-Beam microscope at room temperature, and collected into adhesive caps of collecting tubes. Dissected material was snap-frozen in liquid nitrogen and stored at -80°C until RNA isolation. Specific cell types collected over several LCM sessions were pooled together for RNA extraction.

### Advances of the Protocol

– A comprehensive LCM protocol for the developing xylem of both angio- and gymnosperms, applicable to tangential, radial and cross sections.
– Less laborious protocol, avoiding long embedding procedures common in several published protocols.
– Replaces membrane-coated slides with glass microscope slides, and introduces the easy assembly of a “glass sandwich” to keep anatomical integrity and flatness of the xylem sections.
– Enables isolation of treachery elements, fibers and ray cells for RNA extraction.
– Enables isolation of cell-type specific, good quality total RNA for high-throughput analyses.

## Stepwise Procedures

**FIGURE 1:**
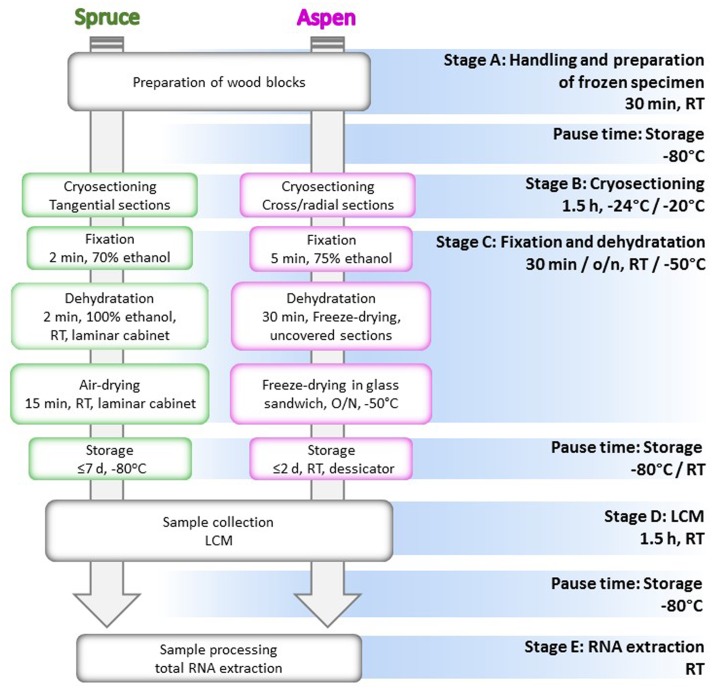
**Flow chart of the cryosectioning and microdissection protocol**. All the stages of the protocol are indicated with the relative temperature and time of realization. Steps performed differently for Norway spruce and aspen are, respectively, included in green and magenta boxes. RT, room temperature; o/n, overnight.

The method is divided into five stages from A to E: (A) tissue sampling, handling and preparation of the frozen specimens; (B) cryosectioning; (C) fixation and dehydration; (D) LCM; (E) RNA extraction and quality assessment (**Figure 1**). We adjusted some of the steps of the protocol taking into account different features of the two woody materials, in order to establish the easiest and fastest technical practice. The goal was to extract good quality RNA from the LCM-dissected cells, and hence, special care was taken to reduce the risk of RNA degradation during each step of the protocol.

### Stage A. Tissue Sampling, Handling, and Preparation of the Frozen Specimens

**Time**: 30 min

**Temperature**: room temperature, or in liquid nitrogen

#### Norway Spruce

A 40-year-old spruce tree was felled during active secondary growth. Disks of the trunk were sawn and immediately frozen in dry ice, and then stored in plastic bags at -80°C.

**FIGURE 2:**
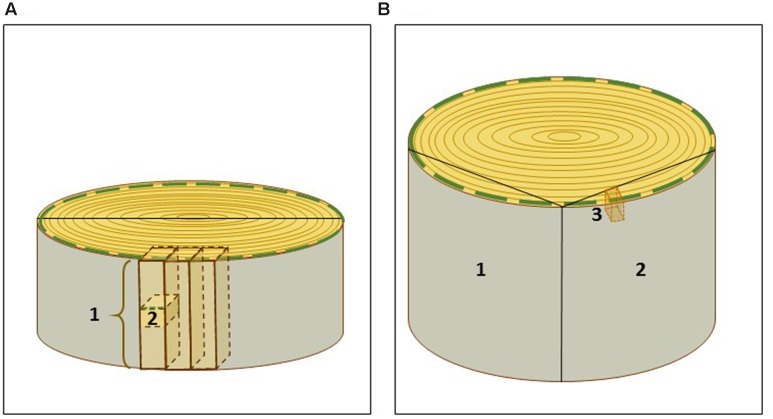
**Scheme of sampling of spruce and aspen wood specimens**. **(A)** Spruce trunk disk was sawn in halves and then, for convenience, in tangential strips of ca. 10.0 cm × 1.2 cm × 1.2 cm (1). Small blocks (2) were cut from the strips and used for cryosectioning. **(B)** Aspen wood samples (1, 2) of ca 30 cm × 20 cm × 5 cm containing bark, current year’s growth and several older annual growth rings, were sawn of wood disks. (3) A small aspen wood block used for cryosectioning. The green dashed line marks the current year’s growth zone used for laser capture microdissection.

In order to avoid thawing and to facilitate the subsequent preparation of cryosections, small wood blocks were cut from the frozen stem disks with a jigsaw. For convenience, the stem disks were first sawn in halves. Further sawing was conducted in two parts: first, a long strip of wood, around 10.0 cm × 1.2 cm × 1.2 cm (H × W × D), was sawn tangentially from the half stem disk (**Figure 2A**). The strip and the remaining stem piece were immediately returned to liquid nitrogen. During this first step the bark was easily removed along the expanding zone of the developing xylem, and thus, developing xylem with secondary cell wall formation on top of the mature xylem was already exposed for cryosectioning. During the second step, the frozen strip of wood was immobilized in a vice and cubic pieces of ca. 1.2 cm × 1.2 cm × 1.2 cm were sawn, placed into 50 ml tubes in liquid nitrogen. The first and the last cubes from the strip were discarded, since a likely thawing in these outermost zones of the strip could compromise the anatomy and the RNA integrity. The cubes were stored at -80°C, and used for cryosectioning within 7 days.

Developing xylem could be identified on the tangential side of the woody cube as a white, almost translucent layer, as opposed to the yellowish, previous year’s growth ring. This facilitated the orientation of the cube during cryosectioning. Alternatively, the side of the mature xylem in the cube was marked red by a quick dipping in 0.5% Safranin O (in 50% ethanol), before storage at -80°C.

#### Aspen

Aspen wood specimens were collected from a 40-year-old tree according to the scheme illustrated in **Figure 2B**. The samples (ca. 30 cm ×20 cm × 5 cm) containing bark, current year’s growth and several older annual growth rings, were cut from the trunk with a jigsaw, snap-frozen in liquid nitrogen and stored at -80°C. Smaller wood blocks, approximately of 3 cm ×1 cm ×3 cm, were cut from the frozen wood material with a jigsaw, and kept in liquid nitrogen before immediate cryosectioning. Alternatively, they were stored at -80°C in aluminum foil, for a longer period. In aspen, the bark was not removed, since it protected the developing xylem during the storage. The bark also helped to orient cutting during sectioning.

##### Key notes for stage A

For collection of wood samples, different strategies of cutting can be applied (**Figure 2**). The samples should be placed to liquid nitrogen as quickly as possible after cutting of smaller pieces to avoid thawing. We also recommend to store the frozen wood specimen at -80°C in small pieces suitable for a single experiment. This minimizes the risk of thawing, and consequently limits the risk of RNA degradation.

During the preparation of the small wood blocks, we advise to define a reference to easily identify the developing xylem zone, for example, by staining as in the case of spruce, or by keeping the bark in place as we did for aspen.

##### Equipment and reagents

Jigsaw; plastic bags; aluminum foil; 50 ml tubes; dry ice; liquid nitrogen; 0.5% Safranin O in 50% ethanol).

### Stage B. Cryosectioning

**Time**: 1.5 h**Temperature**: -20°/-24°C

#### Defining the Direction of Sectioning

**FIGURE 3:**
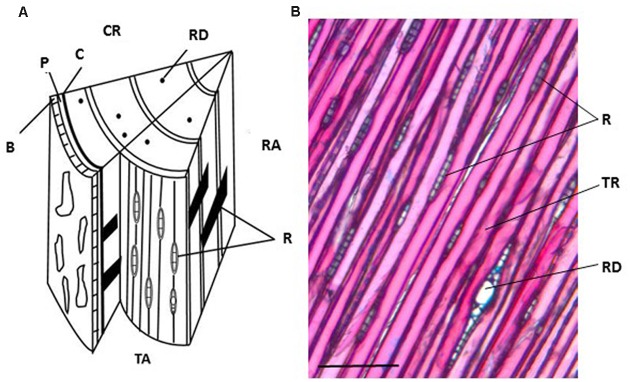
**Anatomy of a Norway spruce stem with cross, radial and tangential sections**. **(A)** Schematic representation of the three sectioning planes in a spruce wood block. **(B)** Tangential section stained with Alcian blue and Safranin *O*. CR, cross section; RA, radial section; TA, tangential section. R, ray cell; B, bark; P, phloem; C, vascular cambium; RD, resin duct; TR, tracheid. Scale bar 150 μm.

The orientation of the block determines the direction of sectioning (**Figure 3A**). **Table 1** summarizes advantages and disadvantages of preparing tangential, cross or radial sections. Tangential sections offer the best view to isolate different cell types, in particular ray cells (**Figure 3**). However, a cross section is necessary to define the thickness of the developing xylem. In the case of aspen, the tangential sections tend to crack along the longitudinal cell walls between fibers and vessels, and hence, cross and radial sections were preferred.

**Table 1:** Choice of the direction of sectioning.

Section orientation	Advantages	Disadvantages
Tangential	Easy isolation of pure ray cell lines. Easy to prepare cryosections, as fibers/tracheids go along the cutting plane.	Difficult to identify the boundaries between the annual growth rings during sectioning. A preliminary estimation of the thickness of developing xylem is required. In hardwoods, the lignified cell walls of fibers/vessels cause longitudinal cracks in the section, and hamper the adequate flatness of the sections on a glass slide.

Radial	Easy to identify fibers and ray cells, and isolate them in large amounts. If the section is perfectly radial (not oblique), the orientation of fibers/tracheids/vessels helps to keep the section flat.	If the section is not perfectly radial, the direction of fibers/tracheids/vessels makes it difficult to fix the sample on the slide and keep it flat. Contamination from the surrounding fiber/tracheid cells is not excluded.

Cross	Easy to distinguish the developing xylem from the mature xylem, and to localize different types of cells. The porous appearance of the section facilitates the laser capture microdissection cut.	When isolating ray cells, contamination from the surrounding fiber/tracheid cells cannot be excluded.

#### Preparation of Tools for Cryosectioning

Glass slides were acid-washed in 1 M HCl for 16 h at c. 55°C. After several washing steps in dH_2_O, the slides were soaked in 100% ethanol, air-dried on a rack and sterilized at 180°C for 4 h. Alternatively, the slides were first washed with RNase decontamination solution or 0.1% DEPC (diethyl pyrocarbonate)-treated water, then with 100% ethanol and finally autoclaved. As a third alternative treatment to remove RNase contamination, the glass slides can also be incubated in a sterile Petri dish under UV radiation for 30 min.

Prior to cryosectioning all the tools, including tweezers and razor blades, were cleaned with the RNase decontamination solution. We do not recommend the use of this solution to decontaminate the cryomicrotome, which can be cleaned with 100% ethanol instead. The temperature of the cryomicrotome was set to -20°C, at least 1 h before starting sectioning. During the first hour of pre-cooling, mounting disks and glass slides were kept in sterile Petri dishes, and together with tweezers, razor blades, and ethanol solutions cooled inside the cryomicrotome chamber.

Membrane-coated slides used in other published protocols (Abbott et al., 2010; Larisch et al., 2012; Zithao et al., 2014), were originally developed for tissue sections of animals, and have been successfully adapted for soft plant tissues. We discovered that glass slides worked better for developing xylem as discussed in more detail under “critical notes”.

#### Estimation of the Thickness of Developing Xylem in Spruce

**FIGURE 4:**
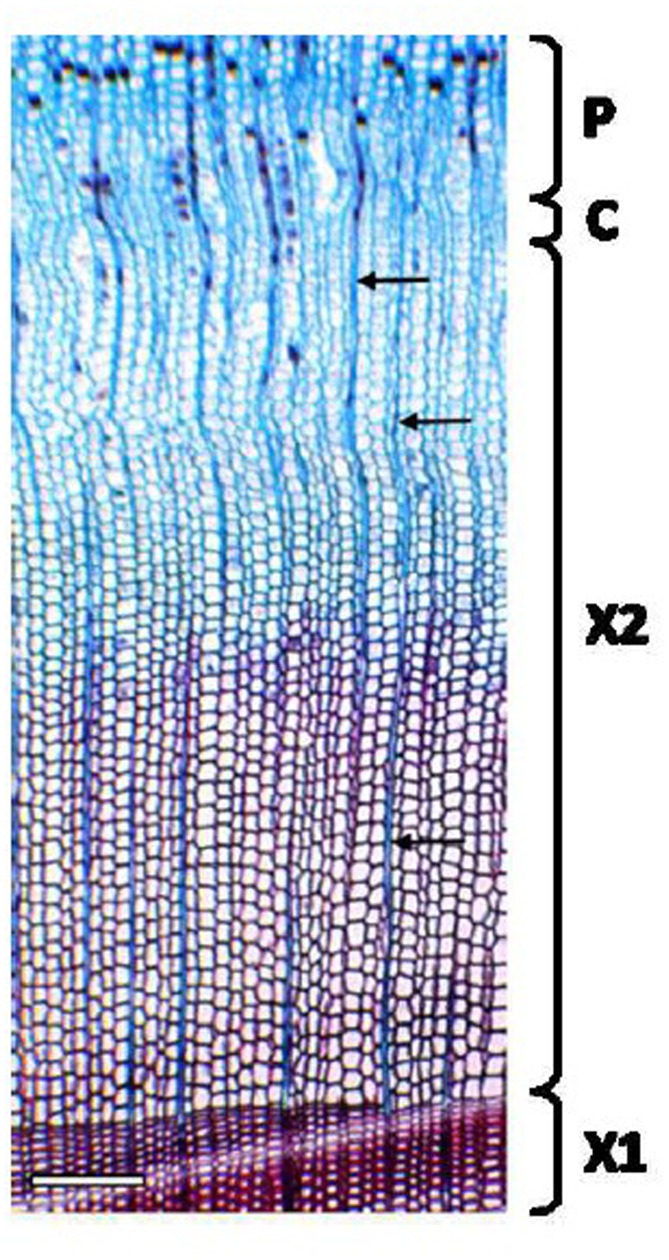
**Cross section of Norway spruce xylem for the estimation of the developing xylem thickness**. A 20 μm thick cryosection was stained with Safranin *O*-Alcian Blue solution. Non-lignified cell walls in developing xylem stain blue with Alcian blue (the outer part of X2). Lignifying cells (the inner part of X2) and the previous year’s growth ring (X1) with fully lignified cell walls stain red with Safranin *O*. Note narrow files of ray cells (arrows). P, phloem; C, cambium; X1, mature, previous year’s xylem; X2, current year’s xylem. Scale bar 250 μm.

The thickness of developing xylem in spruce was determined using an ocular micrometer from 20 μm thick cross sections stained with Safranin *O*-Alcian blue solution (**Figure 4**). The developing xylem layer used in our study was approximately 600 μm thick. Unlignified cell walls stain blue with Alcian blue, and partially lignified cell walls show some red staining with Safranin *O*. Fully lignified cell walls from the previous year’s growth ring appear red under a bright-field microscope.

#### Preparation of Tangential Sections of Spruce

The block of developing xylem was mounted with tweezers on a cooled mounting disk with the tissue freezing medium. We first performed a trimming step, cutting a series of 20 μm thick sections, to remove about 100 μm of the outermost layer of developing xylem. Then, 40 μm thick sections were cut and placed on pre-cooled, RNase-free glass slides. Each slide carried 3–4 sections. According to the previous estimation of the thickness of the developing xylem, the cutting depth did not exceed 600 μm. To facilitate the attachment of the sections to the slide, the slide was slightly warmed from the back with a gloved finger at the site of the sections for ca. 10 s. This allowed collection of the freshly cut sections directly from the cryomicrotome stage, and avoided any further handling or damaging of the sections with tweezers.

#### Preparation of Cross and Radial Sections of Aspen

**FIGURE 5:**
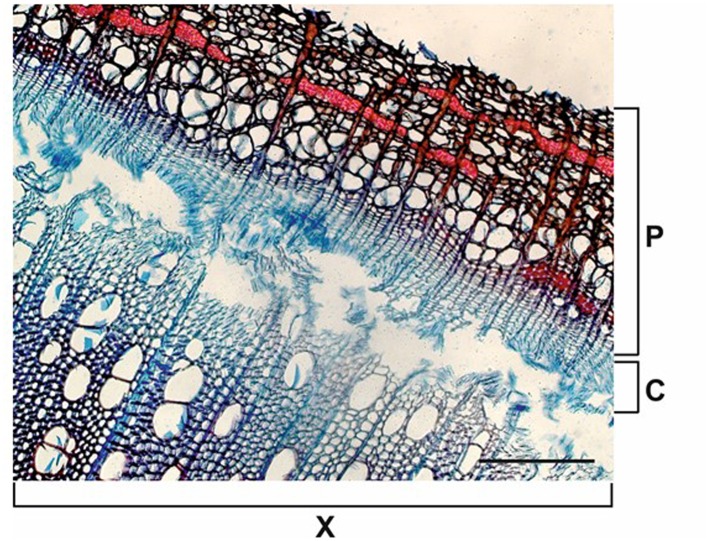
**Cross section of aspen xylem**. A manual cross section of aspen wood was cut during the initial tangential trimming step of cryosectioning. Around 500–700 μm of the phloem (P) was left as a protection and orientation reference during the preparation of cross/radial aspen sections. C, cambial zone; X, current year’s xylem. Scale bar 200 μm.

The frozen wood block was mounted as described above for spruce. Bark and the oldest layers of secondary phloem were removed by cutting serial, 20 μm thick tangential sections. The youngest layers of the secondary phloem, 500–700 μm from the cambium, were left intact to facilitate the cross- and radial-sectioning, and to provide a reference for the orientation of the sections (**Figure 5**). The measurement of the thickness of the bark and the developing xylem was done on cross sections as described above for spruce samples. Cross sections were cut manually with a razor blade from the upper side of the wood block during the trimming step before changing the direction of sectioning. To check the correct orientation and position of the wood sample on the cryotome stage, the first few cryosections were stained with Safranin *O*-Alcian blue, and viewed under a bright field microscope (**Figure 5**).

Three or four 40 μm thick cross or radial sections (around 1 cm × 1 cm/1 cm × 1.5 cm) were cut and delicately positioned using sterile tweezers in 50 μl of 75% ice-cold ethanol on a pre-cooled, RNase-free microscope slide. The addition of ethanol helped the sections to adhere to the slide. The glass slide was kept uncovered and transferred into a 50 ml tube, which was previously cooled in liquid nitrogen. The tube carrying the slide was closed tightly, snap-frozen in liquid nitrogen and kept on dry ice until the freeze-drying step.

##### Key notes for stage B

This stage of the protocol implies the longest handling time for the samples before the fixation. For this reason, appropriate measures should be taken to limit the risk of RNA degradation. Tools and equipment need to be sterile or treated with a RNase decontamination solution or, if not possible, with ethanol. Moreover, the whole procedure has to be done quickly and at low temperature. We recommend to keep all the tools, e.g., razor blades, tweezers, mounting slides, in the cryomicrotome chamber.

##### Equipment and reagents

Cryomicrotome (Leica CM3050 S); UV Crosslinker (Spectrolinker XL-1000); mounting disks; freezing mounting medium (Leica Tissue Freezing Medium, Ref. 14020108926); microscope glass slides; glass Petri dishes; 50 ml tubes; tweezers; razor blades; liquid nitrogen; RNaseZAP^®^ decontamination solution (AMBION AM9780); 100% ethanol; 1 M HCl; 0.1% DEPC (diethyl pyrocarbonate; Sigma–Aldrich^®^, 1609-47-8)-treated water; 75% ethanol solution in RNase-free water (AMBION AM9930); solution (1:1, v/v) of 0.5% Safranin *O* (Merck) in 50% ethanol and 0.5% Alcian blue (National diagnostics) in 50% ethanol.

### Stage C. Ethanol Fixation and Dehydration of the Cryosections

**Time**: 20 min (spruce); 30 min and overnight (aspen)

**Temperature**: room temperature (spruce); room temperature and -50°C (aspen)

#### Fixation and Drying of the Spruce Tangential Sections with Ethanol

Two minutes after the last section was placed onto the slide, ice-cold 70% ethanol was pipetted in excess on top of the sections to fully cover the whole area. The slide was then transferred in a sterile glass Petri dish into a laminar flow cabinet, where 70% ethanol was replaced with ice-cold 100% ethanol. The subsequent fixation step was performed at room temperature in the laminar flow cabinet. During the 2-min incubation in 100% ethanol, the sections were gently stretched and flattened with a pipette tip (see critical notes). After the removal of ethanol by pipetting, sections were air-dried for 15 min. Slides with dry sections were collected into sterile 50 ml tubes. The tubes were tightly closed and stored at -80°C for no longer than 7 days.

#### Fixation and Dehydration by Freeze-Drying of Aspen Samples

**FIGURE 6:**
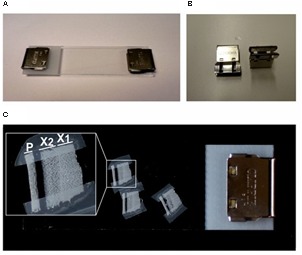
**Glass sandwich assembled for aspen sample preparation**. **(A)** The glass sandwich holding the woody cryosections is closed with a pair of office clips **(B)** for the overnight freeze-drying procedure. **(C)** The freeze-dried sections are attached with tape on the mounting slide and protected in a re-assembled glass sandwich closed by a single clip. In the close-up box, an example of dried cryosection containing an intact xylem zone suitable for laser capture microdissection. P, phloem; X1, old xylem; X2, current year’s xylem.

The freeze-dryer chamber was first cleaned with the RNase decontamination solution and pre-cooled to -50°C. The cryosections were dried as follows: (i) in a closed 50 ml tube for 20 min. The tube cap was kept loose but not totally unscrewed; (ii) in the totally opened tube for additional 5–10 min; (iii) in a closed glass sandwich kept in the 50 ml tube, overnight. The glass sandwich was prepared by covering the glass microscope slide carrying the sections with a second one and closing them on their short sides with a pair of clips (**Figures 6A,B**).

##### Key notes for stage C

This stage is particularly important to obtain flat and dry sections required for successful LCM and good-quality RNA extraction (see critical notes). The glass sandwich keeps the specimens flat during and after drying. However, we observed that the sections were optimal for LCM if they were first kept 30 min uncovered in the freeze-dryer. During this incubation, sections curl and become partially detached from the glass slide, but are still attached all along the phloem and the cambium. After this initial drying step the sections can be flattened without cracks by closing the glass sandwich for the following overnight dehydration.

##### Equipment and reagents

Spruce: ice-cold 70% ethanol in RNase-free water (QIAGEN); 100% ethanol; glass slides; pipette tips; 50 ml tubes; laminar flow cabinet; Aspen: freeze-dryer (Scanvac CoolSafe^TM^), RNaseZAP^®^ decontamination solution (AMBION AM9780); 50 ml tubes; microscope glass slides; office clips.

### Stage D. Laser Capture Microdissection

**Time**: 1.5 h per slide

**Temperature**: room temperature

Laser capture microdissection step was performed similarly for spruce and aspen sections. The only difference was that the aspen samples required an additional preparatory step to immobilize the sections on glass slides. At the end of the overnight freeze-drying, the glass sandwich was removed from the freeze-dryer and delicately opened in the laminar flow cabinet. The dried sections were stably attached to the glass with strips of tape on their longer sides (**Figure 6C**). The glass sandwich was closed again, using now only one clip to avoid disturbance and damage to the sections. Samples were kept in sterile Petri dishes in a portable desiccator chamber, at room temperature until LCM.

The spruce samples were transported to the LCM apparatus in a 50 ml tube kept in liquid nitrogen. Prior to LCM, the tube containing the spruce cryosections was kept at room temperature for 20 min to prevent condensation onto the slide.

**FIGURE 7:**
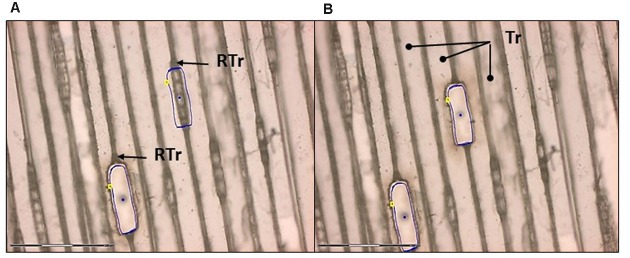
**Laser capture microdissection of ray cells from a tangential section of Norway spruce**. **(A)** Ray parenchymal cells are outlined (blue line) for laser cutting. Note that ray tracheids (RTr) were not collected. One cut and one uncut file of ray cells are seen. **(B)** Ray cells were successfully cut and catapulted. Dots in the middle of the cut regions are the areas where the catapulting pulse was applied. Tr, tracheid. Scale bar 150 μm.

**FIGURE 8:**
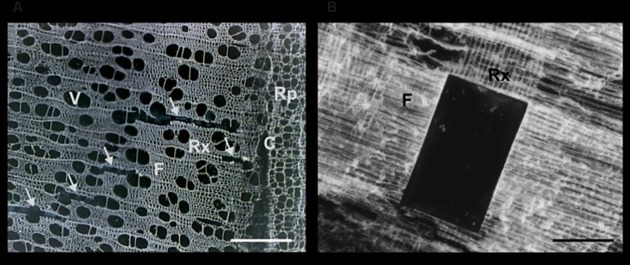
**Sections of aspen wood observed during a LCM session**. The anatomy of wood is well preserved in cross and radial sections of 40 μm thickness. **(A)** In the cross section the empty areas marked by arrows correspond to cut and catapulted ray cells. **(B)** A part of the ray-enriched area of a radial section was cut and catapulted. Rx, xylem ray cells; F, fibers; V, vessels; Rp, phloem ray cells; C, cambium. Scale bar 200 μm.

The specimens were dissected using the PALM^®^ MicroBeam System (Carl Zeiss AB). Optimal laser dissection was achieved with both types of wood according to the following settings: cut speed 20–30; cut energy 60–65; laser power catapulting energy 25–30. The best focus was achieved in the range of 65–70 with delta focus at –2. The optimal magnification for outlining and cutting was 20× (spruce) and 10× (aspen). This is similar to the most suitable magnification for catapulting (20× for spruce and 10× for aspen) (**Figures 7** and **8**).

The dissected pieces were collected by catapulting into the adhesive cap of a 500 μl collecting tube. Each tube and each slide were used during a single LMC session for no longer than 1.5 h. In this time, we were able to catapult around 15–25 and 200–300 microdissected pieces for aspen and Norway spruce, respectively, which generally corresponded to a total area of 0.5–0.75 mm^2^ and 0.2–0.4 mm^2^ of aspen and spruce wood tissue, respectively, collected in a single adhesive cap. Specimens, dissected during several LCM sessions, were combined to obtain a detectable amount of extracted RNA. For example, the total area of spruce LCM-isolated ray parenchymal cells was of ca. 12 mm^2^. At the end of every session (usually 1.5 h long), the collecting tube was snap-frozen in liquid nitrogen. All the dissected samples were stored at -80°C until RNA isolation.

#### Key Notes

We suggest to always prepare more than one slide with 3–4 sections for one LCM session in order to have enough sections in case one appears unsuitable for LCM. The use of RNase-free tools (tweezers and needles) during addition of tape to the freeze-dried sections is recommended although the sections are now fixed, and thus, more protected against RNA degradation. In a troubleshooting **Table 2**, we list the most relevant and common problems faced during LCM.

**Table 2:** Troubleshooting list for LCM and practical recommendations.

Problem	Possible reason	Way out
No or very low amount of RNA detected.	RNA degradation during the section preparation or during the LCM session. Not enough dissected cells.	Improve the procedures for RNase decontamination. Shorten the time at room temperature before or during the LCM session. Increase the number of dissected cells for RNA extraction.

Section sticks to the slide, catapulting of dissected cells is impossible.	Section is not dry enough.	Prolong air-drying or freeze-drying step.

Burning of the section during cutting.	Laser power is too high. Section is too thick.	Reduce laser power. Decrease the thickness. Do not exceed 60 μm in case of hardwood sections.

Loss of power cut during cutting.	The section area is out-of-focus or became out-of-focus during the cut. Oblique sectioning during preparation of cryosections.	Repeat the cut along the outline. Adjust the focus slightly until the laser beam turns thinner and effective again. Improve the orientation of the wood block during sectioning.

Wavy, not perfectly flat section.	Oblique sectioning during preparation of the cryosections. Section not perfectly dry. Section not stably attached to the mounting slide.	Improve the orientation of the wood block during sectioning. Prolong the dehydration step. Avoid condensation onto the slide. Keep the slides in a desiccator chamber. After freeze-drying, add tape to the section more tightly.

Cracked section after the freeze-drying step	Glass sandwich was assembled too early.	Wait longer before assembling the glass sandwich. Slightly increase the section thickness.

#### Equipment and Reagents

Laser microdissection microscope (PALM MicroBeam, Zeiss); adhesive cap tubes 500 μl opaque (Carl Zeiss AB, 415190-9201-000); liquid nitrogen; 50 ml tubes; additionally for aspen: tweezers; needles; office transparent tape (Magic^TM^ invisible 3M, Scotch^®^); scissors; desiccator.

### Stage E. RNA Isolation and Assessment of RNA Quality

**Time**: 2 h, or according to the RNA extraction kit manufacturer’s instructions

**Temperature**: room temperature and according to the RNA extraction kit manufacturer’s instructions

RNA was extracted from the whole cryosections and dissected cells pooled from several LCM sessions. Total RNA quality was assessed using the RNA Pico Assay for 2100 Bioanalyzer.

**FIGURE 9:**
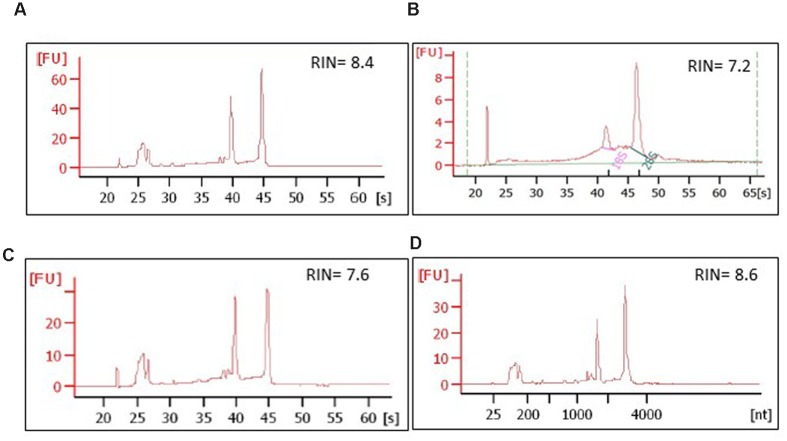
**Integrity of RNA isolated from aspen and spruce cryosections and from the laser-dissected material**. The RNA quality was assessed at every step of the protocol, on: **(A)** a whole, freeze-dried cross cryosection, aspen; **(B)** microdissected ray parenchymal cells, spruce; **(C)** an aspen cross cryosection from which ray cells were laser-dissected; the material, in this case, was snap-frozen in liquid nitrogen after the LCM session and stored at -80°C for 2 days; **(D)** intact aspen sections, not used for LCM, but left at room temperature in a desiccator chamber for 2 days prior to RNA extraction. [s], migration time, seconds; [nt], nucleotide size; RIN, RNA integrity number.

Aspen sections required a longer preparation time than those of spruce, and were consequently more susceptible to RNA integrity impairment, especially during the last pre-LCM stage at room temperature. Total RNA quality was therefore measured at every step of the protocol, i.e., in a single intact cryosection, in a single intact cryosection fixed and left at room temperature in the desiccator for two days, in microdissected cells, and in residual sections used for LCM (**Figures 9A,C,D**). In all these samples the RIN (RNA Integrity Number) was between 6 and 9 confirming that no significant RNA degradation occurred during the whole procedure.

In spruce, the RNA quality was assessed first in whole cryosections. Twenty sections, each 20 μm thick, were pooled and processed in the same way as microdissected samples, i.e., fixed and dehydrated in ethanol series, and then incubated for 2 h at room temperature, equal to the time of the LCM session. As a control, an identical set of whole cryosections were kept at -80°C for no longer than 7 days and analyzed. No significant impairment of the RNA integrity was observed in the sections stored at room temperature. The quality of total RNA isolated from the separately dissected tracheids and ray parenchymal cells was of adequate quality for RNA sequencing (RIN 6–9, **Figure 9B**).

#### Key Notes

**FIGURE 10:**
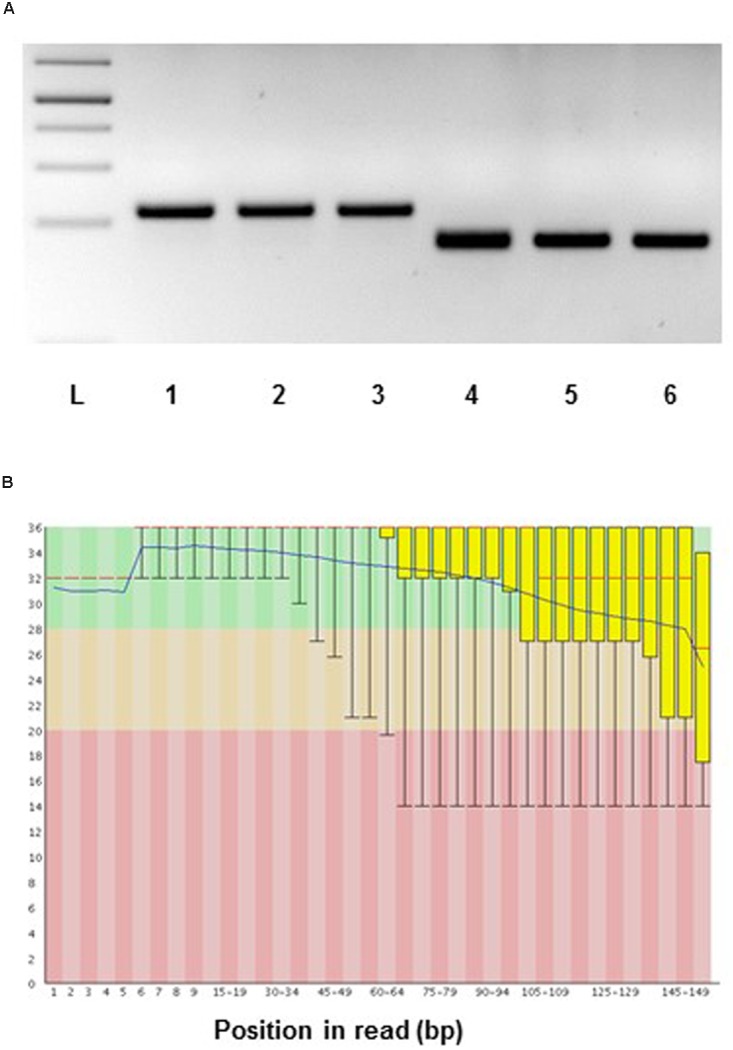
**Suitability of RNA from LCM samples for gene expression analysis**. **(A)** Aspen: reverse transcriptase-PCR of laser microdissected developing wood fibers of *Populus tremula*. RNA was amplified using the MessageAmp^TM^ II aRNA kit (ThermoFischer) and then subjected to RT-PCR using actin and ubiquitin specific primers. PCR templates: 1 and 4, cDNA library constructed from *Populus tremula x tremuloides* developing wood total RNA; 2 and 5, cDNA library constructed from laser microdissected aspen fiber cells (starting from 4 ng of RNA); 3 and 6, cDNA library constructed from laser microdissected fiber cells (starting from 6 ng of RNA). PCR primers: 1 to 3, *ACTIN*; 4 to 6, *UBIQUITIN*. L, 1 kb DNA ladder. PCR 35 cycles. **(B)** RNA sequencing, spruce: representative quality scores across all bases (Sanger/Illumina 1.9 encoding) for xylem tracheids collected by LCM. Y-axis – quality scores; the higher the score the better the base call. Green area – very good quality calls, orange – reasonable quality calls, red – calls of poor quality. Red line – median value, blue line – mean quality.

The RNA yield we could obtain with our protocol was for spruce ray parenchymal cells and xylem tracheids c. 60 ng, and for the whole sections from 90 to 180 ng. In the case of aspen, the total RNA yield measured on LCM dissected samples was between 5 and 20 ng on average for xylem fibers and rays. The RNA yield per volume of the tissue was 0.5 ng/mm^3^ (spruce whole sections), 3.36 ng/mm^3^ (spruce tracheids), 9.6 ng/mm^3^ (spruce ray cells), and 0.7–2.1 ng/mm^3^ (aspen ray cells). To test the suitability of aspen samples for transcript analysis, two LCM samples containing approximately 4 and 6 ng RNA was amplified and subjected to reverse transcriptase-PCR (RT-PCR) using actin and ubiquitin primers. RT-PCR products were detected on an agarose gel, a cDNA library from total developing wood was used as a positive control (**Figure 10A**). For spruce we have successfully performed RNA sequencing on the Illumina platform for ray parenchymal cells, tracheids, and for whole xylem sections (**Figure 10B**). The transcriptomic data which were aligned to the Norway spruce genome (Nystedt et al., 2013), have been submitted to the European Nucleotide Archive ENA (Study ID PRJEB10305/ERP011536, to be released when the work will be published).

#### Equipment and Reagents

RNA extraction kit for LCM material/small samples (RNAqueous^®^ Micro Kit, AMBION) (aspen); RNeasy^®^ Plant Mini Kit (QIAGEN, 74904) (spruce); RNase free water (AMBION AM9930 (aspen); QIAGEN (spruce)); RNA assay kit (AGILENT RNA 6000 PICO KIT 5067-1513); Agilent 2100 G2938A Bioanalyzer for RNA quantitation and quality assessment. MessageAmp^TM^ II aRNA kit (ThermoFisher (aspen)); RT-PCR kit (Thermofischer Maxima First Strand cDNA Synthesis Kit for RT-qPCR, with dsDNase); actin and ubiquitin primers (aspen): ACT-F: TGAGGATAT TCAGCCCCTTG, ACT-R: CCATGCTCAATGGGGTATTT, UBI-F: AGATGTGCTGTTCATGTTGTCC, UBI-R: ACAGCCACTCCAAACAGTACC.

#### Critical Notes

##### Thickness of the sections

The presence of the cell wall in plant tissues poses a challenge in laser microdissection of plant samples. Lignification of secondary cell walls adds another challenge in LCM of woody samples. Softer plant tissues, such as developing phloem, can be cut by LCM from 100 μm thick sections (Jyske et al., 2015), but no individual cell types can be isolated. We experienced that very thin sections, 20 μm thick, do not allow extraction of detectable amounts of RNA, while LCM was unable to cut sections exceeding 60 μm without visible burning/charring of the sample. We could successfully microdissect individual cell types of developing xylem, using 30 and 40 μm thick sections. In the case of aspen, 40 μm of thickness represented the best choice to achieve histological integrity, efficient LCM and good quality RNA extraction. Aspen cryosections thinner than 40 μm were easier to cut and catapult, but were more fragile, and thus, showed cracking more frequently after the dehydration step.

##### Flatness of the sections

In LCM the target cells are visualized *via* light microscopy. This requires preparation of flat sections on the mounting slide in order to avoid undesirable out-of-focus blur. Improper focusing of the specimen does not only disturb accurate identification of the target cells, but also prevents proper laser beam focusing during the cut.

The strategies of sectioning and fixation selected in our protocol helped to optimize the flatness of the sections and the overall quality of the plant samples. In contrast to the majority of the protocols on LCM of plant tissues (Takahashi et al., 2010; Rajhi et al., 2011; Shiono et al., 2014), we excluded the paraffin-embedding procedure. Paraffin-embedding of aspen samples was tested, but was found to damage the anatomy and cause RNA degradation. Hence, cryosectioning as reported previously (Cañas et al., 2014; Jyske et al., 2015) was the preferred choice. During cryosectioning the size and the orientation of the blocks were extremely important. To facilitate the handling of the sections during fixation (**Figure 1**, stage C) and to improve the flatness of the sections, the section size should not exceed 1.5 cm × 1.5 cm. Moreover, as summarized in **Table 1**, the most appropriate direction for sectioning was the one facilitating the visualization of specific cell types of interest and guaranteeing the highest integrity of the dry sections.

During the fixation step, xylem sections do not stably adhere on the membrane-coated slides. PEN membrane slides are widely used in LCM of animal and some plant tissues (Jyske et al., 2015), but, in our case, their adhesive force was not effective enough to obtain flat sections, probably because of the thickness and the hard structure of our woody sections. This problem was overcome by replacing the PEN slides with plain glass slides, and applying two different approaches for spruce and aspen. On one hand, adequately flat sections of developing spruce xylem were obtained by gently flattening the sections with a pipette tip. This was the most efficient method, especially because it was fast and minimized the manipulation of the sections. For aspen, we designed a glass sandwich method to prepare flat and intact xylem sections. The specimen was dry and no significant RNA degradation was detected in sections stored for two days in a desiccator at room temperature (**Figure 9D**).

##### Avoiding RNA degradation

The goal of our protocol was to extract good quality total RNA from specific cell types of developing xylem of spruce and aspen. The protocol was developed to limit RNase contamination and RNA degradation during sampling and further processing of the samples. We prevented RNase contamination by working with sterile instruments, by cleaning all the tools and equipment with RNase decontamination solution or with ethanol, and using UV radiation to pretreat glass slides. RNA degradation was minimized by limiting the handling of the wood blocks and the sections at room temperature prior to the fixation step. Similarly, we minimized the exposure of spruce sections at room temperature during fixation, applying a fast procedure based on two sequential ethanol incubations, the first of which was performed in the cryomicrotome chamber at -24°C. Fixed spruce sections were stored at -80°C for no longer than 7 days, and good quality RNA was extracted (RIN higher than 7) from the sections (**Figure 9B**). In the case of aspen, the total dehydration of the ethanol-treated sections, performed by freeze-drying, was an effective alternative approach for fixation. Keeping the freeze-dried sections at room temperature, in a desiccator chamber for 2 days, did not decrease the RNA quality. Finally, LCM sessions lasted no longer than 1.5 h per slide. This was critical to prevent undesired RNA degradation.

## Concluding Remarks

The protocol described here allows isolation of ray parenchymal cells, tracheary elements, and fibers from developing xylem of both angiosperm and gymnosperm tree species by combining optimized techniques of wood section preparation and LCM technology. The resulting RNA is of high quality demonstrating that the protocol can be applied to transcriptome analyses aimed at deciphering cell-specific molecular events.

## Author Contributions

OB and CV contributed to the concept and design of the work, performed the experiments and wrote the manuscript; KS performed experiments and wrote the manuscript; LZ performed experiments and was involved in drafting the manuscript; AK, TN, and KF substantially contributed to the concept and design of the experiments and took part in writing of the manuscript.

## Conflict of Interest Statement

The authors declare that the research was conducted in the absence of any commercial or financial relationships that could be construed as a potential conflict of interest.

## References

[B1] Abbott E. Hall D. Hamberger B. Bohlmann J. (2010). Laser microdissection of conifer stem tissues: isolation and analysis of high quality RNA, terpene synthase enzyme activity and terpenoid metabolites from resin ducts and cambial zone tissue of white spruce (*Picea glauca*). *BMC Plant Biol.* 10:106 10.1186/1471-2229-10-106 PMC3095273 20540781

[B2] Asano T. Masumura T. Kusano H. Kikuchi S. Kurita A. Shimada H. (2002). Construction of a specialized cDNA library from plant cells isolated by laser capture microdissection: toward comprehensive analysis of genes expressed in the rice phloem. *Plant J.* 32 401–408. 10.1046/j.1365-313X.2002.01423.x 12410817

[B3] Cañas R. A. Canales J. Gómez-Maldonado J. Ávila C. Cánovas F. M. (2014). Transcriptome analysis in maritime pine using laser capture microdissection and 454 pyrosequencing. *Tree Physiol.* 34 1278–1288. 10.1093/treephys/tpt113 24391165

[B4] Gaude N. Bortfeld S. Duensing N. Lohse M. Krajinsk F. (2012). Arbuscule-containing and non-colonized cortical cells of mycorrhizal roots undergo extensive and specific reprogramming during arbuscular mycorrhizal development. *Plant J.* 69 510–528. 10.1111/j.1365-313X.2011.04810.x 21978245

[B5] Gautam V. Sarkar A. K. (2015). Laser assisted microdissection, an efficient technique to understand tissue specific gene expression pattern and functional genomics in plants. *Mol. Biotechnol.* 57 299–308. 10.1007/s12033-014-9824-3 25403993

[B6] Gomez K. S. Javot H. Deewatthanawong P. Torres-Jerez I. Tang Y. Blancaflor E. B. (2009). *Medicago truncatula* and *Glomus intraradices* gene expression in cortical cells harboring arbuscules in the arbuscular mycorrhizal symbiosis. *BMC Plant Biol.* 9:10 10.1186/1471-2229-9-10 PMC2649119 19161626

[B7] Goué N. Lesage-Descauses M.-C. Mellerowicz E. J. Magel E. Label P. Sundberg B. (2008). Microgenomic analysis reveals cell type-specific gene expression patterns between ray and fusiform initials within the cambial meristem of *Populus*. *New Phytol.* 180 45–56. 10.1111/j.1469-8137.2008.02556.x 18631289

[B8] Goué N. Noël-Boizot N. Vallance M. Magel E. Label P. (2012). Microdissection to isolate vascular cambium cells in poplar. *Silva Fennica* 46 5–16.

[B9] Hogekamp C. Arndt D. Pereira P. A. Becker J. D. Hohnjec N. Küster H. (2011). Laser microdissection unravels cell-type-specific transcription in arbuscular mycorrhizal roots, including CAAT-box transcription factor gene expression correlating with fungal contact and spread. *Plant Physiol.* 157 2023–2043. 10.1104/pp.111.186635 22034628 PMC3327204

[B10] Horneffer V. Linz N. Vogel A. (2007). Principles of laser-induced separation and transport of living cells. *J. Biomed. Opt.* 12:054016 10.1117/1.2799194 17994904

[B11] Inada N. Wildermuth M. C. (2005). Novel tissue preparation method and cell-specific marker for laser microdissection of *Arabidopsis* mature leaf. *Planta* 221 9–16. 10.1007/s00425-004-1427-y 15578216

[B12] Jardinaud M.-F. Boivin S. Rodde N. Catrice O. Kisiala A. Lepage A. (2016). A laser dissection-RNAseq analysis highlights the activation of cytokinin pathways by nod factors in the *Medicago truncatula* root epidermis. *Plant Physiol.* 171 2256–2276. 10.1104/pp.16.00711 27217496 PMC4936592

[B13] Jyske T. M. Suuronen J. P. Pranovich A. V. Laakso T. Watanabe U. Kuroda K. (2015). Seasonal variation in formation, structure, and chemical properties of phloem in *Picea abies* as studied by novel microtechniques. *Planta* 242 613–629. 10.1007/s00425-015-2347-8 26105650

[B14] Kerk N. M. Ceserani T. Tausta S. L. Sussex I. M. Nelson T. M. (2003). Laser capture microdissection of cells from plant tissues. *Plant Physiol.* 132 27–35. 10.1104/pp.102.018127 12746508 PMC1540312

[B15] Larisch C. Dittrich M. Wildhagen H. Lautner S. Fromm J. Polle A. (2012). Poplar wood rays are involved in seasonal remodeling of tree physiology. *Plant Physiol.* 160 1515–1529. 10.1104/pp.112.202291 22992511 PMC3490584

[B16] Nagy N. E. Sikora K. Krokene P. Hietala A. M. Solheim H. Fossdal C. G. (2014). Using laser micro-dissection and qRT-PCR to analyze cell type-specific gene expression in Norway spruce phloem. *PeerJ* 2:e362 10.7717/peerj.362 PMC4017884 24860697

[B17] Nakazono M. Qiu F. Borsuk L. A. Schnable P. S. (2003). Laser-capture microdissection, a tool for the global analysis of gene expression in specific plant cell types: identification of genes expressed differentially in epidermal cells or vascular tissues of maize. *Plant Cell* 15 583–596. 10.1105/tpc.008102 12615934 PMC150015

[B18] Nystedt B. Street N. R. Wetterbom A. Zuccolo A. Lin Y. C. Scofield D. G. (2013). The Norway spruce genome sequence and conifer genome evolution. *Nature* 497 579–584. 10.1038/nature12211 23698360

[B19] Rajhi I. Yamauch T. Takahashi H. Nishiuchi S. Shiono K. Watanabe R. (2011). Identification of genes expressed in maize root cortical cells during lysigenous aerenchyma formation using laser microdissection and microarray analyses. *New Phytol.* 190 351–368. 10.1111/j.1469-8137.2010.03535.x 21091694

[B20] Roux B. Rodde N. Jardinaud M.-F. Timmers T. Sauviac L. Cottret L. (2014). An integrated analysis of plant and bacterial gene expression in symbiotic root nodules using laser-capture microdissection coupled to RNA sequencing. *Plant J.* 77 817–837. 10.1111/tpj.12442 24483147

[B21] Shiono K. Yamauchi T. Yamazaki S. Mohanty B. Malik A. I. Nagamura Y. (2014). Microarray analysis of laser-microdissected tissues indicates the biosynthesis of suberin in the outer part of roots during formation of a barrier to radial oxygen loss in rice (*Oryza sativa*). *J. Exp. Bot.* 65 4795–4806. 10.1093/jxb/eru235 24913626

[B22] Takahashi H. Kamakura H. Sato Y. Shiono K. Abiko T. Tsutsumi N. (2010). Method for obtaining high quality RNA from paraffin sections of plant tissues by laser microdissection. *J. Plant Res.* 123 807–813. 10.1007/s10265-010-0319-4 20221666

[B23] Zheng P. Aoki D. Matsushita Y. Yagami S. Sano Y. Yoshida M. (2016). Lignification of ray parenchyma cells (RPCs) in the xylem of *Phellodendron amurense* Rupr.: quantitative and structural investigation by TOFSIMS and thioacidolysis of laser microdissection cuts of RPCs. *Holzforschung* 70 641–652. 10.1515/hf-2015-0120

[B24] Zithao L. Kayan O. Tao Y. Hubiao C. Zhongzen Z. (2014). Cell type-specific qualitative and quantitative analysis of saikosaponins in three *Bupleurum* species using laser microdissectionand liquid chromatography–quadrupole/time of flight-massspectrometry. *J. Pharm. Biomed. Anal.* 97 157–165. 10.1016/j.jpba.2014.04.033 24863374

